# Characteristics and Outcome of Children and Adolescents Diagnosed With Ewing Sarcoma Treated at a Tertiary Cancer Center in India: A Single-Institution Experience

**DOI:** 10.7759/cureus.77423

**Published:** 2025-01-14

**Authors:** Chinmay Doctor, Dipesh Dave, Maharshi Trivedi, Harsha P Panchal, Abhijeet A Salunke

**Affiliations:** 1 Department of Pediatric Oncology, Gujarat Cancer and Research Institute, Ahmedabad, IND; 2 Department of Medical Oncology, Gujarat Cancer and Research Institute, Ahmedabad, IND; 3 Department of Surgical Oncology, Gujarat Cancer and Research Institute, Ahmedabad, IND

**Keywords:** adolescent cancers, bone cancer, chemotherapy for ewing sarcoma, ewing sarcoma, outcomes of ewing sarcoma

## Abstract

Introduction: Ewing sarcoma (ES) is a malignant, aggressive tumor most frequently diagnosed among pediatric and adolescent populations. Patients with ES in low-middle income countries (LMIC) have dismal outcomes. The aim of this study is to evaluate the outcomes in children and adolescents with ES treated using a multimodal uniform treatment protocol.

Methods: A retrospective analysis was conducted on 54 children and adolescents <18 years of age diagnosed with ES who received treatment from January 2021 to December 2022 at a tertiary cancer center in Western India. Data were retrieved from the hospital database, including clinical and medical records.

Results: The study included 32 males (59%) and 22 females (41%) with a male-to-female ratio of 1.45:1. The median age of presentation was 13 years (ranging from four to 17 years). Localized disease was observed in 36 patients (67%) and metastatic disease in 18 patients (33%). Extremities were the most common primary sites (35 patients, 65%). Among the patients, 15 (28%) achieved complete response (CR), seven (13%) had persistent disease, 25 (46%) had incomplete response, and seven (13%) experienced disease progression on chemotherapy. The median (IQR) OS was 36 months (30-42 months), with three-year overall survival (OS) and progression-free survival (PFS) rates of 55% and 40%, respectively.

Conclusion: In ES, complete resection of tumors with negative margins (i.e., R0 resection) significantly improves outcomes when combined with chemotherapy and radiotherapy. Survival is greatly increased upon achieving CR, highlighting the significance of successful early therapy. For this patient population to have better outcomes, further research and closing disparities in healthcare in LMICs are essential.

## Introduction

Ewing sarcoma (ES) is a highly aggressive tumor of bones and soft tissues, predominantly affecting children and adolescents [1]. It is the second most prevalent primary bone cancer in this population [2]. The incidence of primary bone tumors is around nine in 1 million in a year, with ES comprising around 15.9% of these cases [2]. ES peaks between the ages of 10 and 20, primarily affecting people in their first two decades of life with modest male predominance [2]. The hallmark of ES is chromosomal translocation, most commonly t(11;22) (q24;q12). This translocation generates the EWS-FLI1 fusion protein, which acts as a primary driver of oncogenic activity in ES [3]. This distinctive translocation provides targets for diagnosis and treatment and explains the malignant nature of ES.

Multidisciplinary management of ES involves a combination of neoadjuvant and adjuvant chemotherapy, surgical resection of the tumor, and/or radiation therapy based on the stage and location of the tumor [3]. High-dose chemotherapy regimens like the INT-0091 protocol, which include drugs like vincristine, doxorubicin, cyclophosphamide, ifosfamide, and etoposide, have shown better results [4]. Surgical management with wide resection of the tumor remains a keystone in the prognosis of patients with ES [4]. However, more effective methods are needed to enhance survival because the prognosis for patients with high-risk characteristics like large tumor size, pelvic site, or metastasis at diagnosis remains poor, with a five-year overall survival (OS) of around 50% [2,3].

The management of ES in India and other low-middle income countries (LMIC) faces significant challenges such as late presentation due to limited awareness, socioeconomic challenges, limited access to specialized healthcare, lack of specialized tools for diagnosis, and high rate of treatment defaulters [3]. High-income countries (HICs) have advanced healthcare systems and better public health infrastructures, while LMICs face resource constraints, delayed presentation, and financial barriers, leading to poor survival rates and unfavorable outcomes [3,5].

The literature from HICs highlights the importance of early diagnosis and successful multimodal therapy by reporting a higher OS for localized disease and metastatic disease [2]. However, results are frequently less promising in LMICs like India, where multiple limiting factors lead to low overall survival rates [3]. The prognosis is greatly impacted by these circumstances, which frequently cause patients to come with metastasis or advanced stages of the disease at the time of diagnosis.

Thus, the objective of this retrospective study is to assess the complete response (CR) rate, OS, and progression-free survival (PFS) in patients treated with multimodal uniform treatment protocol in children and adolescents with ES.

## Materials and methods

Patient selection and study design

We conducted a retrospective analysis of children and adolescents <18 years of age diagnosed with ES between January 2021 and December 2022 at The Gujarat Cancer and Research Institute, Ahmedabad, India, after receiving approval from the institutional review committee (IRC/2024/P-88). Patients with treatment dropouts and those with insufficient data were excluded from the study. Demographic details, treatment information, response assessment, and outcome were documented. MRI was used to determine the local extent of the staging. Chest computed tomography and bone scan/positron emission tomography scan were done to evaluate lung and bone metastases. A bone marrow biopsy was done to look for bone marrow involvement. Core needle biopsy and immuno-histochemistry staining for FLI1 and CD99 were used to confirm the diagnosis. To measure tumor response, the proportion of necrosis following chemotherapy was assessed in surgical specimens. Treatment response was classified as either CR, partial response, or progressive disease. The PFS, OS, and salvage therapy outcomes were analyzed using the follow-up data.

Treatment protocols

In our institution, all patients diagnosed with ES received chemotherapy (INT-0091 protocol), and surgery was performed in operable patients, post-week-12 chemotherapy when feasible for local disease control, with options ranging from limb-sparing with complete resection and negative margins (i.e., R0 resection) or amputation in certain cases. The radiotherapy was used for residual disease or inoperable tumors (Table 3 in Appendix).

Statistical analysis

Microsoft Excel sheets were used to document the clinical and demographic data of patients. The clinical characteristics and demographics of the patients were summarized using descriptive statistics. A statistical analysis was considered "significant" if the p-value was less than 0.05. The Kaplan-Meier method was used to calculate survival. For this study, SPSS software version 22.0 (IBM Corp., Armonk, USA) was used for analysis.

## Results

Patient demographics and clinical characteristics

The study included 54 children and adolescents diagnosed with ES. Seven patients were excluded due to incomplete data and treatment abandonment. The median age of presentation was 13 years, ranging from four to 17 years. Twenty-seven (50%) were aged between 10 and 15 years, and 10 patients (18%) were older than 15 years. Age distribution revealed that 17 patients (31%) were under 10 years, and 37 patients (69%) were above 10 years of age. The majority of the patients were male, comprising 59% (n=32), while female patients accounted for 41% (n=22), resulting in a male-to-female ratio of 1.45:1 (Table 1).

**Table 1:** Characteristics of patients with Ewing sarcoma treated at a tertiary cancer center

Characteristics	Number of Patients (%)
Gender	
Male	32 (59%)
Female	22 (41%)
Age	
≤ 10 years	17 (31%)
>10 years	37 (69%)
Site of Primary tumor	
Extremities	35 (65%)
Pelvis	12 (22%)
Rib	7 (13%)
Disease at Presentation	
Localized	36 (67%)
Metastatic	18 (33%)

The primary tumor sites included the extremities in 35 patients (65%), the pelvis in 12 patients (22%), and ribs in seven patients (13%). At the time of diagnosis, 36 patients (67%) presented with localized disease, while 18 patients (33%) had metastatic disease. The most common sites of metastasis were the lungs (72%) and bone marrow (28%).

Treatment characteristics

All patients received neoadjuvant and adjuvant chemotherapy following the INT-0091 protocol. Surgical resection was performed in 42 patients (78%), with R0 resection (negative margins) achieved in 41 cases (76%) and R1 resection (microscopic positive margins) in one case (2%). Limb-sparing surgery was feasible in 36 patients (89%) of surgeries, whereas six (11%) patients underwent amputations due to extensive disease or late presentation. Radiotherapy was administered to 13 patients (24%), one due to R1 disease and 12 as definitive radiotherapy (Table 2).

**Table 2:** Characteristics and response to treatment of patients with Ewing sarcoma treated at a tertiary cancer center

Characteristics	Number (%)
Chemotherapy	54 (100%)
Surgery (n=42)	
Limb-sparing surgery	36 (67%)
Amputations	6 (11%)
Radiotherapy	13 (24%)
Response to Treatment	
Complete response	15 (28%)
Partial response	25 (46%)
Stable disease	7 (13%)
Progressive disease	7 (13%)
Survival Analysis	
Median Overall survival (95% CI)	30-42 months
Median progression free survival (95% CI)	18-30 months
Relapsed Patients	18 (33%)
Second-line salvage Chemotherapy	12 (22%)
Palliative care	6 (11%)
Survival for second-line treatment	
Median progression-free survival (95% CI)	6 to 18 months

Response to treatment

Post-treatment evaluations revealed that 15 patients (28%) achieved CR, 25 patients (46%) had a partial response (PR), seven patients (13%) showed stable disease (SD), and seven patients (13%) had progressive disease (PD).

Survival analysis

**Figure 1:**
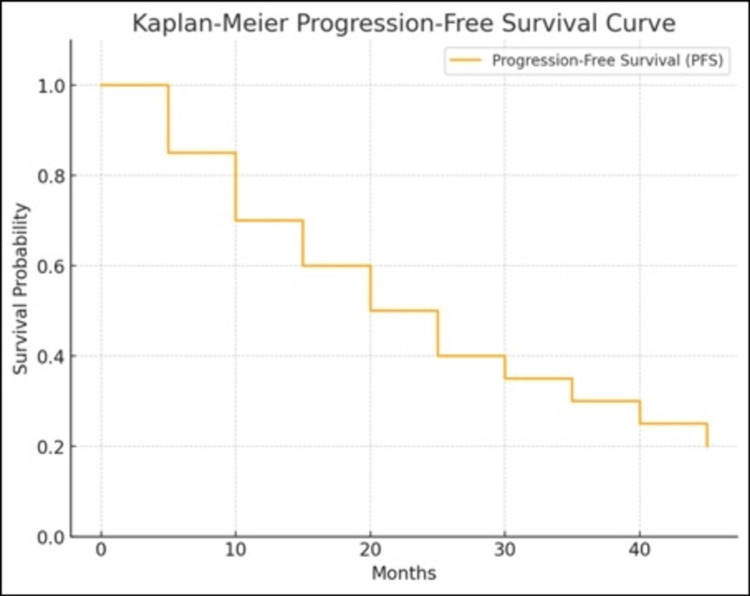
Kaplan- Meier PFS curve of patients with Ewing sarcoma treated at tertiary cancer center PFS: Progression Free Survival

The median follow-up duration was 24 months (range 12-48 months). PFS was conducted using the Kaplan-Meier method, revealing a median PFS of 24 months (95% confidence interval: 18-30 months) (Figure 1). The three-year PFS rate was observed at 40%.

**Figure 2:**
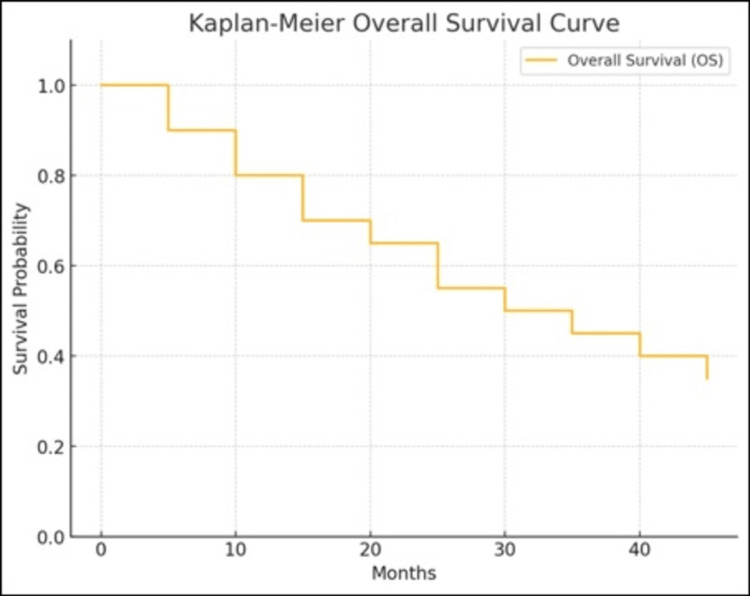
Kaplan- Meier OS curve of patients with Ewing sarcoma treated at tertiary cancer center OS: Overall Survival

The median OS was 36 months (95% confidence interval: 30-42 months), with a three-year OS rate of 55% (Figure 2).

Among 54 children and adolescent patients, 18 (33%) patients relapsed on follow-up. Among those, 12 received various salvage chemotherapy as a second-line chemotherapy and six patients were transitioned to palliative care. The median PFS throughout the second-line treatment was 12 months, with a 95% confidence interval ranging from six to 18 months.

## Discussion

ES constitutes 3-4% of all cancers in children and adolescents [1]. The outcomes have improved over the period because of the multimodal approach [1]. The present study analyses the clinical features, strategies for treatment, and outcomes of ES patients in children and adolescents who received medical care at a tertiary cancer center. In order to maximize survival in this aggressive cancer, the results highlight the significance of a multidisciplinary strategy that combines systemic chemotherapy, surgery, and radiation therapy.

The survival outcome is poor in the LMIC environment because of delayed presentation at diagnosis, inadequate disease knowledge and awareness, restricted access to healthcare, treatment abandonment, and lost to follow-up [3,5]. A key component of managing ES is surgical resection, which, when paired with chemotherapy and radiation therapy, improves survival and provides local control. Since limb-sparing surgery preserves function without sacrificing oncological results, it has emerged as the favored method [6]. However, the viability of the best surgical procedures is frequently constrained in LMICs by a lack of resources and delayed diagnoses [5].

In their research, Sasi et al. found that 40.3% of patients had metastases at the time of diagnosis; the current study found that 33% of patients had metastases [7]. According to the results of our studies, metastatic ES at presentation still has a dismal prognosis. With over 33% of patients having metastases at presentation and nearly 13% progressing after treatment. We found results similar to the observation by Batra et al. that metastatic disease, large size of tumor and unresponsiveness to chemotherapy have inferior OS [8]. Compared to the current study, higher survival rates have been observed in the studies from the Western world [9]. Nevertheless, only 27.2% of patients in their study developed metastatic disease, compared to 33% in the current analysis [9]. In the current study, the OS is approximately 36 months, with a three-year OS rate of 55%. Batra et al. in their study mentioned 25% of patients were metastatic at presentation and five-year OS in their study was 45.1% [8]. Pant et al. in their study showed 42.3% OS with the highest percentage of patients from the extremities [10].

The prognosis for children and adolescents with ES is poor, and although salvage chemotherapy can help with symptoms, it is still very difficult to achieve long-term results [11]. Nearly 33% of patients in the current research experienced a relapse at follow-up, with a median PFS of 12 months during second-line treatment, with a 95% CI of six to 18 months. In their study, Grier et al. found that adding etoposide and ifosfamide to the usual regimen for ES significantly improved the result for patients with ES who did not have metastases [4]. According to the current study, these regimens had an overall OS of about 55%.

Shukla et al. describe the oncologic and demographic consequences of upper extremity ES in a subset of the Indian population [6]. The survival rate for patients with upper extremity ES who underwent limb salvage surgery was similar to that of those who underwent amputation surgery [6]. The results of Tiwari et al. suggest that ES in Indian patients were shown to be worse than those described in the Western research, despite the fact that their study's demographic profile, stage at presentation, and local and systemic therapy regimen were comparable to those discovered in the global literature [5].

Based on the study's findings, a prompt and accurate assessment of ES is crucial for initiating effective and successful treatment, which has various implications for clinical practice. The primary objective of treatment must be to achieve CR because of its strong link with improved survival rates. Because of the limited efficacy of second-line drugs, individuals with ES who have relapsed or demonstrated treatment resistance require novel therapeutic strategies.

The strength of the study was that all patients were treated using the uniform treatment protocol at a single center and there were no major protocol deviations. Limitations of our study are a small sample size and retrospective design. Moreover, this study includes data from a single regional center, which might not be applicable to the larger population from different geographic regions. These limitations can be overcome by conducting a prospective study or trial involving multiple centers from different regions.

## Conclusions

We conclude that children and adolescents with ES in LMIC still have a moderate prognosis even after receiving intense multimodal therapy. Patients who could achieve CR attained better outcomes compared to the patients who could not achieve CR.

## Appendices

**Table 3:** Institutional treatment protocol for children and adolescents with Ewing sarcoma V- Vincristine;  2 mg/m^2^ intravenous (IV) push. A- Doxorubicin; bolus infusion at a dose of 75mg/ m^2^ on day 1 and day 2. C- C- Cyclophosphamide; dose of 1200 mg/m^2^. M- Mesna; 360 mg/m^2^ at 0, 4, 8 hours after cyclophosphamide/ifosfamide. I- Ifosfamide; 1800 mg/m^2^ from day 1 to day 5 IV over 1 hour. E- Etoposide; 100 mg/m^2^ from day 1 to day 5 IV over 1 hour. a- Actinomycin D; 1.25 mg/m^2^. A cumulative dose of adriamycin should not exceed 375 mg/m^2^. Pegylated granulocyte stimulating factor (PEG-GCSF) at a dose of 0.1 mg/kg was given 24 hours post-chemotherapy.

Week	Chemotherapy Drugs	
0	V, A, C, M	
3	I, E, M	
6	V, A, C , M	
9	I, E, M	
12	V, A, C , M	
EVALUATION		
15	I, E, M	Surgery and/ or Radiotherapy
18	I, E, M	
21	I, E, M	
24	V, A, C , M	
27	I, E, M	
30	V, a, C, M	
33	I, E, M	
36	V, a, C, M	
39	I, E, M	
42	V, a, C, M	
45	I, E, M	
48	I, E, M	
